# Preserving the rectus femoris and improving limb function after total femoral prosthesis replacement following resection of femoral malignant tumors

**DOI:** 10.3389/fonc.2023.1149342

**Published:** 2023-03-14

**Authors:** Fan Wu, Xiang Fang, Dechao Yuan, Yan Xiong, Yi Luo, Wenli Zhang, Chongqi Tu, Hong Duan

**Affiliations:** ^1^ Department of Orthopedics, West China School of Medicine/West China Hospital, Sichuan University, Chengdu, China; ^2^ Department of Orthopedics, Zigong Fourth People’s Hospital, Zigong, China

**Keywords:** femur, tumor, total femoral prosthesis replacement, limb-salvage, rectus femoris invasion

## Abstract

**Background:**

Current research is focused on the factors that influence the maintenance of limb function after total femoral replacement. This retrospective study investigated the difference in functional outcomes in patients with invasion of the rectus femoris *vs*. an intact rectus femoris that underwent total femoral replacement with a modular total femur prosthesis.

**Methods:**

The medical records of patients who underwent total femoral replacement with a modular total femur prosthesis between July 2010 and March 2017 at our institute were retrospectively reviewed. The patients were divided into two groups: group A had invasion of the rectus femoris and group B had an intact rectus femoris. Functional status was assessed using the Musculoskeletal Tumor Society Rating Scale (MSTS) and the Harris Hip Score (HHS). Complications were assessed using the International Society of Limb Salvage classification that was published in 2011 and modified in 2014.

**Results:**

The mean total MSTS score (23.0 ± 4.8 *vs*. 17.6 ± 3.1; *P* = 0.02) and the mean total HHS score (80.17 ± 6.24 *vs*. 55.38 ± 13.30; *P* = 0.001) were significantly higher in patients with intact rectus femoris compared with patients with invasion of the rectus femoris. Patients with an intact rectus femoris achieved significantly better limb function (support and gait) and active range of motion (*P* < 0.05). The overall complication rate was 35.7%.

**Conclusions:**

Functional outcomes after total femoral replacement were significantly better in patients with an intact rectus femoris compared with patients with invasion of the rectus femoris, possibly because more muscle mass can be preserved around the femur in patients with an intact rectus femoris.

## 1 Introduction

The femur is commonly affected by primary and secondary malignant bone tumors that require radical surgical excision in the lower extremities (1). The primary purpose of the treatment is to save the patient’s life. A patient with a femur malignant tumor has a very poor prognosis, and until 1972, the survival rates ranged from 5% to 20% (2), and before the 1980s, amputation was the only treatment. Furthermore, the survival rate of the patients is not improved by amputation (3), the limb function is not good, and the psychological trauma is profound. With the advancement of surgical techniques, implant designs, diagnostic imaging systems, and chemotherapy methods, not only has the survival rate increased significantly but also limb salvage after tumor resection has become a standard approach and flourished. To date, the 5-year survival rate of osteosarcoma has been reported to be between 65% and 86% (4).

When tumors have extensive involvement and have multiple or skip lesions and when previous distal or proximal replacement failed, the treatment is quite difficult, and in such instances, total femur replacement (TFR) is recommended (5). Hip disarticulation, turnabout procedure (6), and tibial turn-up procedure (7) are alternative surgical approaches. The prosthesis includes a metallic system procedure (8) and a total femoral allograft (9). Considering its mature use and accessibility, a metallic system prosthesis is most commonly used in the clinic. TFR can restore the integrity of the femur and allow the patients to resume ambulation pain-free, and the function of TFR was much better than hip dislocation (2) and turnabout and turn-up procedures (7, 10). In addition, limb salvage is the expectation of most patients.

TFR requires a great sacrifice for the affected muscles. The extent of quadriceps removal has been reported to influence the long-term functional efficiency of a patient’s gait, and the function of patients who have had reserved the rectus femoris after total knee replacement for treating the distal femoral tumors is better than the function of those who had not reserved the rectus femoris (11). Theoretically, the rectus femoris is the only muscle in the quadriceps that spans from the hip to the knee joint, and the function is to bend the hip and extend the knee; preserving the rectus femoris in total femur replacement had a better hip and knee function similar to total knee replacement.

To counteract or prevent the factors that contribute to the limitation of the hip and knee functions after TFR, it may prove valuable to reserve the rectus femoris to increase hip and knee function. We performed a retrospective cohort study to determine whether there are differences between a TFR with and without the rectus femoris invasion. We sought to conclude 1) the effect of total femur replacement and 2) whether patients without invasion of the rectus femoris had a better hip and knee range of motion (ROM) or a better function.

## 2 Materials and methods

We retrospectively reviewed 14 patients with total femoral prosthesis replacement between July 2010 and March 2017 at our institute. There were eight men and six women, with ages ranging from 16 to 75 years (average age of 44.8 years). There were 11 cases of primary tumors and 3 cases of metastatic tumors. The origin of the primary cases was as follows: three involved the diaphysis, two the distal third of the femur, four the long segment of the shaft, and two the proximal third of the femur; three metastases involved the long segment of the femur (one caused pathological fracture) which were secondary to lung cancer. All patients had more than one segment of the femur involved, which required a total femur replacement, as retaining any part of the femur for proximal, shaft, or distal prostheses would have been inappropriate and unstable. The most common diagnosis in these cases was osteosarcoma (eight cases). The patients were divided into two groups: group A had invasion of the rectus femoris (eight cases), and group B had an intact rectus femoris (six cases) (**Table 1**). This study cohort was approved by the Ethics Committee of West China Hospital of Sichuan University, and all the participants were informed about the surgical approaches.

**Table 1:** Clinical characteristics of the included patients.

Number	Age (years)/gender	Diagnosis	Rectus femoris invasion	MSTS	HHS	Follow-up time (months)
1	75/M	Metastatic tumor	No	22	75	Alive, 17
2	54/M	Metastatic tumor	No	18	76	Alive, 14
3	47/F	Osteosarcoma	Yes	23	76	Dead, 12
4	28/F	Osteosarcoma	Yes	15	44	Dead, 16
5	25/F	Osteosarcoma	Yes	18	52	Dead, 13
6	62/M	Osteosarcoma	Yes	16	36	Dead, 11
7	68/M	Chondrosarcoma	No	28	81	Alive, 58
8	73/M	Osteosarcoma	No	21	74	Alive, 18
9	16/F	Osteosarcoma	No	29	90	Alive, 27
10	32/F	Chondrosarcoma	No	20	83	Alive, 32
11	17/M	Osteosarcoma	Yes	16	51	Alive, 18
12	19/M	Osteosarcoma	No	21	69	Alive, 43
13	42/F	Chondrosarcoma	Yes	18	64	Alive, 12
14	72/M	Metastatic tumor	Yes	14	51	Dead, 15

HSS, Harris Hip Score; MSTS, Musculoskeletal Tumor Society Rating Scale.

### 2.1 Surgical procedures

Preoperative systematic evaluations and examinations, including clinical evaluations, plain radiographs, single-photon emission computerized tomography (SPECT) scans, chest radiographs, and computed tomography (CT) scans, were performed to assess local lesions and metastases. Magnetic resonance imaging (MRI) was used to determine the extent of tumor invasion, including the involvement of soft tissue, especially neurovascular tissue.

All the patients with osteosarcoma received preoperative and postoperative chemotherapies with high-dose methotrexate, doxorubicin, cisplatin, and ifosfamide. The patients with femur pathological fractures following metastasis tumor received targeted drugs and comprehensive treatment for the primary tumor.

The patients received a modular total femur prosthesis (Chunlizhengda Co. Ltd., Beijing, China), which contains a bipolar femoral head component and a fixed hinge, cemented, and constrained total knee system.

**Figure 1:**
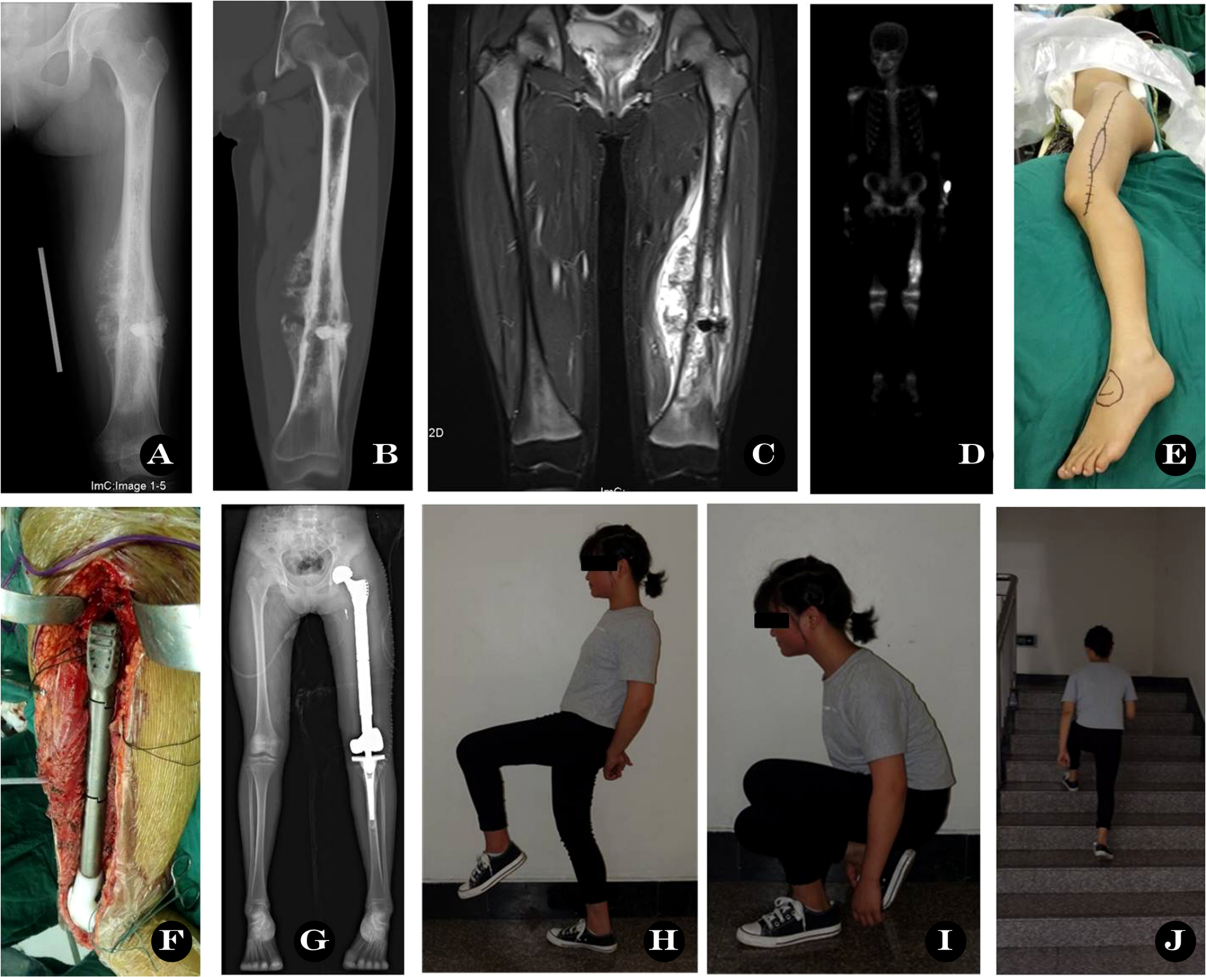
Total femoral prosthesis replacement. A 15-year old girl with left femur osteosarcoma. **(A)** X-ray before operation, **(B–D)** CT, MRI and SPECT image showing massive involvement, **(E)** Surgical incision, **(F)** Total femur prosthesis in operation, **(G)** X-ray post-operation, **(H–J)** Functional outcome at 33 months.

Surgery was performed using the long lateral approach to the femur (**Figure 1E**) and involved three steps. After a long incision was made on the lateral side from 10 cm proximal to the greater trochanter to the anterolateral aspect of the patellar tendon and tibial tuberosity, en-bloc excision of the entire femur was performed. At the proximal thigh, the gluteus medius, gluteus minimus, and deep external rotators were detached depending on the surgical margin (5, 12). The gluteus maximus tendon was separated, and the sciatic nerve and vascular bundles were exposed and well protected. The hip capsule was dissected around the femoral neck, and the femoral head was dislocated. At the distal thigh, the patella was turned to the medial dislocation; the neurovascular bundles were separated from the tumor, exposed, and protected well; the adductor muscles were separated and the muscles attached to the linea aspera were removed; the capsule at the knee was divided; and then the total femur was removed. The tumor is resected according to the principles (13), endeavoring to achieve a satisfactory resection margin while the lesion was completely removed. A transverse osteotomy was performed 10 mm below the tibial joint line to allow the cementation of the tibial component. The second main step was the reconstruction of the defect with a prosthesis. The proximal tibia was osteotomized and the tibia component was inserted and then the cement was fixed. The femoral components were assembled, and the stability and tension were tested. Finally, in the third main step, the tissue was reconstructed. The remaining hip capsule was fixed around the neck of the prosthesis, and the external rotation muscles were sutured to the repaired capsule to strengthen. The remaining psoas muscle was rotated forward and was sutured on the capsule. The remaining abductor muscles will be placed on the proximal side of the prosthesis and reattached to the metal ring or the remaining greater trochanter, an artificial ligament was needed if these structures were not sufficient. The concept of the principle of the tumor-free technique was very important. Careful hemostasis was crucial, and dead space was eliminated as much as possible. When the wound was sutured, the prosthesis was covered with the rest of the muscles, and the wound was sutured in layers, with an indwelling drainage tube. If necessary, the vascularized gastrocnemius muscle flap blood vessels were used to cover the wound. All patients used an abduction brace after surgery. All patients received intravenous broad-spectrum antibiotics before and after the surgery.

Patients with total femoral replacement can gain full independence through a comprehensive and adequate rehabilitation program. Physical therapy techniques such as muscle contraction, passive and active exercises, and isometric exercises were very useful during early rehabilitation. Certain exercises, such as active hip abduction or knee flexion, were permitted 3-4 weeks later to protect the muscles which have been reattached to the prosthesis. Partial weight-bearing was allowed 6 weeks later. After 8~12 weeks, patients were advised to practice walking with a single crutch to determine whether their walking gait has normalized or not.

Patients were followed up every month in the first 3 months, every 3 months for the first year, and then every 6 months. A chest CT scan was performed every 3 months during the first year and then every 6 months for patients with osteosarcoma. A SPECT bone scan was performed every 6 months in the first year, then once a year, until the last follow-up.

### 2.2 Outcome measures

Functional status was assessed using the Musculoskeletal Tumor Society Rating Scale (MSTS) (14) and the Harris Hip Score (HHS) at the last follow-up. The MSTS constitutes six items (pain, function, emotional acceptance, use of an external support, walking ability, gait alteration) scored on a scale of 0 to 5 to a maximum of 30, with higher scores indicating better function. The HHS constitutes 10 items in domains that include pain, function, absence of deformity, and ROM, scored to a maximum of 100 with higher scores indicating better function. Complications were assessed using the International Society of Limb Salvage classification that was published in 2011 (15) and modified in 2014 (16). Type I is soft tissue failure, type II is aseptic loosening with clinical and radiographic signs of loosening, type III is structural failure, type IV is periprosthetic infection requiring removal and subsequent reimplantation of the implant, and type V is tumor progression (16).

### 2.3 Statistical analyses

Statistical analyses were performed with the SPSS software package version 24.0 (SPSS Inc., Chicago, IL, USA). Groups were compared using independent *t*-tests and the chi-square test for the continuous and categorical variables, respectively. A *P*-value <0.05 was considered significant.

## 3 Results

The demographic and clinical characteristics of the included patients stratified by invasion of the rectus femoris (group A) or an intact rectus femoris (group B) are summarized in **Table 2**. There were no significant differences in age (*P* = 0.27), gender (*P* = 0.569), diagnosis (*P* = 0.486), operative time (*P* = 0.759), blood loss (*P* = 0.59), or follow-up period (*P* = 0.182) between the two groups.

**Table 2:** Demographic and clinical characteristics of the included patients stratified by invasion of the rectus femoris or an intact rectus femoris.

	Invasion of the rectus femoris	Intact rectus femoris	*P*-value	95% CI
Age	39.0 ± 20.3	52.7 ± 23.9	0.270	−12.10, 39.44
Gender			0.569	−0.79, 0.45
Male	4	4		
Female	4	2		
Follow-up (months)	17.5 ± 10.6	27.7 ± 16.3	0.182	−5.46, 25.79
Operative time (min)	235.0 ± 40.1	240.8 ± 24.2	0.759	−34.60, 46.27
Blood loss (ml)	1225.0 ± 710	1008.3 ± 743.2	0.590	−1,069.06, 635.73
Diagnosis			0.486	
Osteosarcoma	5	3		
Chondrosarcoma	2	1		
Metastatic tumor	1	2		

The functional results of patients with invasion of the rectus femoris (group A) and an intact rectus femoris (group B) are summarized in **Table 3**. The mean total MSTS score was 66.4% (19.93/30). The mean total MSTS score was significantly higher in patients with an intact rectus femoris (23.0 ± 4.8) compared with patients with invasion of the rectus femoris (17.6 ± 3.1) (*P* = 0.02). Specifically, patients with an intact rectus femoris scored significantly better on the function (*P* = 0.04), support (*P* = 0.003), and gait (*P* = 0.016) items of the MSTS, but there were no significant differences in the pain (*P* = 0.20), emotional acceptance (*P* = 0.802), or walking (*P* = 0.178) items between the two groups. The mean total HHS score was significantly higher in patients with an intact rectus femoris (80.17 ± 6.24) compared with patients with invasion of the rectus femoris (55.38 ± 13.30) (*P* = 0.001). Specifically, patients with an intact rectus femoris scored significantly better in the pain (*P* = 0.003), function (*P* = 0.001), and ROM (*P* = 0.026) domains of the HHS, but there was no significant difference in the deformity domain between the two groups (*P* = 0.433).

**Table 3:** Functional outcomes of the included patients stratified by invasion of the rectus femoris or an intact rectus femoris.

	Invasion of the rectus femoris	Intact rectus femoris	*P-*value	95% CI
MSTS				
Total score	17.6 ± 3.1	23.0 ± 4.8	0.02	1.00, 9.75
Pain	3.38 ± 0.52	3.83 ± 0.75	0.20	−0.28, 1.20
Function	2.75 ± 0.71	3.67 ± 0.82	0.04	0.03, 1.81
Emotional acceptance	3.75 ± 0.46	3.83 ± 0.75	0.802	−0.62, 0.79
Supporting	2.38 ± 0.74	4.00 ± 0.89	0.003	0.67, 2.58
Walking	2.63 ± 1.06	3.50 ± 1.2	0.178	−0.46, 2.21
Gait	2.63 ± 1.06	4.00 ± 0.63	0.016	0.31, 2.44
HHS				
Total score	55.38 ± 13.30	80.17 ± 6.24	0.001	11.92, 37.66
Pain	30.50 ± 6.48	40.67 ± 1.63	0.003	4.21, 16.12
Function	17.50 ± 6.68	32.50 ± 5.89	0.001	7.52, 22.49
Deformity	3.63 ± 0.52	3.83 ± 0.41	0.433	−0.35, 0.77
ROM	2.50 ± 0.54	3.17 ± 0.41	0.026	0.10, 1.24

HHS, Harris Hip Score; MSTS, Musculoskeletal Tumor Society Rating Scale; ROM, range of motion.

### 3.1 Complications

In general, the complication rate was 35.7% (5/14). Four patients suffered tumor progression (type V failure), three patients had pulmonary metastases, and one patient had local recurrence and amputation 8 months later. One patient experienced deep venous thrombosis that was resolved with antithrombotic therapy. There was no incidence of superficial or deep infection, sciatic paralysis, hip dislocation, or aseptic loosening.

## 4 Discussion

Total femoral prosthesis replacement is a procedure rarely done, and the indications are not well defined (3). From the first total femur prosthesis reported in 1965, the indications focused mainly on oncology diseases, and many authors applied them to malignant femur tumors (1–3, 17). A high-grade malignant tumor that affected the femur widely or totally, or skip lesions, has been the indication for this procedure, including osteosarcoma, Ewing’s sarcoma, chondrosarcoma (18), undifferentiated sarcoma, huge soft tissue tumor, metastatic tumor, and local recurrent osteosarcoma. Moreover, the applications were developed gradually for non-oncology diseases, which can affect the integrity of the femur and could repeatedly cause pathological fractures, such as Paget’s disease, osteogenesis imperfecta (19), fibrous dysplasia with massive idiopathic osteolysis, massive femoral hemophilic pseudotumor (20), and hydatid disease (21). Here, we summarized the main large case series of femoral prosthesis replacement for oncology in the current literature in **Table 4**. Hip and knee arthroplasty revisions with severe bone defects using conventional methods are difficult procedures, and in severe periprosthetic fractures (31), a second-stage arthroplasty approach to prevent infection is required (32). TFR provides patients with a functional limb and enables patients to remain pain-free for the rest of their lives (3).

**Table 4:** Main large case series of total femur replacement for oncology in the current literature.

Ref.	Publication	*N*	Age	Indications	Follow-up (months)	Patients living at the time of publication	Survivorship	All-cause revision rate	Complications
Ahmed (12)	Arch Orthop Trauma Surg	9	47 (10-74)	Oncology	51 (8-200)	4/9	No failures	0%	Infection (2), tibial component loosening (1)
Mankin et al. (22)	Clin Orthop Relat Res	15	52 ± 1 (16-82)	Oncology (14), non-oncology (1)	54 (12-192)	7/15	NA	33.3%	Infection (1), prosthesis failure (4)
Nerubay et al. (23)	Clin Orthop Relat Res	19	20	Oncology	18-96	7/19	NA	–	Wound-healing problems (10), infection (1), popliteal vein injury (1), prosthesis failure (1)
Steinbrink et al. (24)	J Bone Joint Surg Br	32 (28 patients)	56 (21-81)	Oncology (6), non-oncology (22)	6-84	23/28	NA	9.4%	Infection (2), hip dislocation (1), prosthesis failure (1), patellar pain (1)
Ward et al. (25)	Clin Orthop Relat Res	21	44.6 (11-91)	Oncology (17), non-oncology (4)	31 (1-125)	11/21	NA	2.4%	Infection (3), hip dislocation (2), patellar pain (1)
Sevelda et al. (26)	Clin Orthop Relat Res	11	64 (41-78)	Metastatic carcinoma	5 (1-31)	8/11 died after 6 months	NA	–	Hip dislocation (1), infection (1), local recurrence (1)
Liu et al. (27)	World Journal of Surgical Oncology	21	21.8	Osteosarcoma	71.2	72.5% last follow-up	66.7% at 5 years	–	Superficial infection (2), deep infection (1), patella fracture (1), local recurrence (1), pulmonary metastases (9), tibial stem loosening (3)
Puri et al. (28)	Indian J Orthop	8	32	Osteosarcoma (5), Ewing’s sarcoma (1), chondrosarcoma (2)	33 (9-72)	5/8 (24-72months)	5/8	–	Infection (1), metastasis (1), 3 cm shortening (1)
Jones et al. (29)	J Surg Oncol	54	40.6 ± 19.9	Primary sarcoma (40), metastatic sarcoma (1), metastatic carcinoma (12), lymphoma (1)	48 (1-252)	28/40	28/40	–	Hip dislocation (5), femoral malrotation (1), infection (4)
Muratori (30)	Journal of Orthopaedics	32	54.2 (13-82)	Oncology (23), non-oncology (9)	60	NA	NA	–	Superficial infection (2), deep infection (1), dislocation (2)

NA, not available.

The symbol "-" means that column was not mentioned in the article.

The mean MSTS score and ROM of the 14 patients were 66.4% (19.93/30) and 65.9°, and the mean HHS score of the hip was 66 (44-90). Similar to the results of Sewell et al. (1), the mean MSTS score was 67% and the mean HHS was 70%. However, in contrast to the study of Sewell et al. (1), we did not compare the difference between the primary and the secondary TFRs because our sample size was too small. This function score is generally lower than TKR and THR, but it is acceptable to those tumor patients. Our typical case function is provided in the supplement video (**Figure 1**, **Supplement 1**).

The function after TFR was good for pathological fractures following metastasis. In our three cases with pathological fractures, the function was good (the mean MSTS score was 18). Similar to the report of Mankin et al. (22), involving a total of 15 patients with 2 found to have metastatic carcinomas, the function and quality of life of the survivors were good. Total femoral prosthesis arthroplasty can recover early functional weight-bearing walking and exercise and effectively guarantee improvement in the quality of life of the survivor. However, the resection of the entire femur requires wide exposure and a prolonged procedure time. As the amount of intraoperative blood as well as surgical trauma is large, surgeons must dynamically assess the patient’s heart–lung capability and degree of tolerance. In the study of Sevelda et al. (26), the authors summarized that of the 11 patients with metastatic carcinoma of the femur, 8 of them died 6 months after the operation, so they believe that TFR does not warrant greater life expectancy, and patients with extensive metastatic disease to the femur should be offered palliative care rather than major reconstruction. Thus, an increase in the sample size for TFR for pathological fractures is needed. In **Table 4**, we summarized the main functional outcomes and follow-up results of TFR in the literature.

The rectus femoris is very important in the function of patients with TFR. In our cases, we found that patients without rectus femoris invasion had better limb function (including supporting, walking, and gait) and a greater range of active hip movement than those with rectus femoris invasion. The rectus femoris is the only muscle in the quadriceps that spans from the hip to the knee joint, and its anatomical location is superficial in the quadriceps. Once the rectus femoris of a patient is invaded, then he will have a wider resection to obtain enough surgical boundaries, and fewer muscles around the femur could be preserved. Benedetti et al. (11) reported similar results for total knee arthroplasty in the distal femoral tumor that preserves the rectus femoris. Similar to our findings, Morris et al. (5) proposed that a lack of hip abductors or knee extensor procedures resulted in a poor functional outcome as the patient cannot control the limb. Nakayama et al. (33) reported that the most influential factor in the functional outcomes after TFR was whether the rectus femoris was preserved or not. Du et al. (34) found that the use of an artificial ligament to reconstruct soft tissue can improve limb function after TFR, but this was indirectly confirmed. The mean MSTS function score was 66.4% (19.93/30), similar to the reports by Ahmed (12), Kalra et al. (3), and Du et al. (34). The mean HHS score of the hip was 66 (44-90), similar to the report by Sewell et al. (1), and the overall mean HHS score was 70 (51-86). TFR provides most of the patients with a functional limb, which can be weight-bearing and make walking pain-free.

The reported complication rates vary (35, 36), and we summarized the main complications of TFR in the literature in **Table 4**. In our cases, the complication rate was 35.7% (5/14). Common complications included infection, aseptic failure, hip dislocation, and vascular and sciatic nerve injury. The most frequent complications were dislocation and infection (37, 38). Our cases have no infection mainly because we use an impulse-type flusher and plenty of saline water to wash the wounds. In the report by Kalra et al. (3), the deep infection rate was 7%, which was similar to the report by Mankin et al. (22). While in the report by Natarajan (17), the deep infection rate was 11.8%. Friesecke et al. (38) published the largest known series of total femur prostheses, involving 100 consecutive patients, and the infection rate was 13%. The dislocation rate reported by Friesecke et al. (38) was 6%; however, our cases and the report by Sevelda et al. (39) do not have dislocations. The reason why we had no dislocation was mainly due to the good reconstruction of the hip abductor, and we used artificial ligaments for large defects. Du et al. (34) found that the use of an artificial ligament can decrease the dislocation rate. The greater trochanter and the accompanying outriggers are essential to maintain the stability of the prosthesis, so it is very important to refix these outsoles to the prosthesis.

The current data from a series of patients suggest that TFR plays a role in treating malignant or even severely damaged benign femoral lesions. The death and complication rates are high, which might be due to the degree of malignancy, size, and vascularity of the lesion, but the functional outcome of the survivors is reasonable and better than hip dislocation or hemisection. In metastatic femoral metastases with pathological fractures, the quality of life can be improved within a limited lifetime.

Our study has some limitations. First, the number of cases is small, due to the rare indication for this procedure, although our data cover a span of 8 years. Second, it has a retrospective design, which had some selection bias from the inclusion of non-randomized patients. Third, our cases had different types of tumors, which received an individualized general prognosis because of the rare indications for TFR.

## 5 Conclusion

We believe that our report provides the expected results for patients with femoral tumors who require total femoral replacement. This form of reconstruction provides predictable results after the removal of the femoral tumor. TFR is a good and reliable method for the salvage of the femoral tumor limb.

## Data availability statement

The original contributions presented in the study are included in the article/**Supplementary Material**. Further inquiries can be directed to the corresponding author.

## Ethics statement

The studies involving human participants were reviewed and approved by Ethics Committee of West China Hospital of Sichuan University. Written informed consent to participate in this study was provided by the participants’ legal guardian/next of kin. Written informed consent was obtained from the individual(s) for the publication of any identifiable images or data included in this article.

## Author contributions

FW contributed to the conception and execution of the research and the writing of the manuscript. XF, DY, WZ, CT and HD were responsible for the integrity and analysis of the data. YX and YL contributed to the conception and execution of the research. All authors contributed to the article and approved the submitted version.
